# Evaluation of serum moesin and podocalyxin levels in patients with diabetes mellitus

**DOI:** 10.1515/med-2026-1421

**Published:** 2026-04-28

**Authors:** Selin Genc, Oguzhan Aksu, Ummugulsum Can, Hatice Caliskan Burgucu, Selma Ozlem Celikdelen, Jelena Kotur Stevuljevic, Aleksandra Klisic, Enver Ciftel, Filiz Mercantepe

**Affiliations:** Department of Endocrinology and Metabolism, Konya State Hospital, Konya, Türkiye; Department of Biochemistry, Konya State Hospital, Konya, Türkiye; Department of Internal Medicine, Konya State Hospital, Konya, Türkiye; Department for Medical Biochemistry, Faculty of Pharmacy, University of Belgrade, Belgrade, Serbia; Department of Biochemistry, Faculty of Medicine, University of Montenegro, Podgorica, Montenegro; Center for Laboratory Diagnostics, Primary Health Care Center, Podgorica, Montenegro; Department of Endocrinology and Metabolism, Sivas Numune Hospital, Sivas, Türkiye; Department of Endocrinology and Metabolism, Faculty of Medicine, Duzce University, Duzce, Türkiye

**Keywords:** diabetic nephropathy, moesin, podocalyxin, biomarkers, endothelial dysfunction, podocyte injury

## Abstract

**Objectives:**

Diabetic nephropathy (DN) is a major microvascular complication of diabetes and a leading cause of end-stage renal disease. Reliable biomarkers for early DN detection remain limited. Moesin and podocalyxin, involved in endothelial and podocyte integrity, may contribute to DN pathophysiology. This study evaluated whether serum moesin and podocalyxin levels differ according to the presence of early-stage DN in type 2 diabetes.

**Methods:**

In this single-center cross-sectional study, 63 patients with type 2 diabetes and 27 healthy controls were included. Diabetic participants were classified as normoalbuminuric diabetes (DM) or microalbuminuric early DN (DNP) based on spot urine albumin-to-creatinine ratio. Serum moesin and podocalyxin levels were measured by ELISA. Group comparisons and regression analyses were performed.

**Results:**

Both biomarkers were significantly lower in diabetic patients than in controls (p<0.001), but no difference was observed between DM and DNP after age adjustment. Neither biomarker independently predicted nephropathy. A composite factor including moesin, podocalyxin, age, white blood cell count, and microalbuminuria was associated with DN (OR 4.03, 95 % CI 1.42–11.47, p=0.009).

**Conclusions:**

Serum moesin and podocalyxin decrease in type 2 diabetes but do not independently distinguish early DN, suggesting limited kidney-specific utility.

## Introduction

Diabetic kidney disease, also referred to as diabetic nephropathy (DN), is one of the most important microvascular complications of diabetes (DM) and remains a leading cause of end-stage renal disease worldwide [1], [2], [3]. Current guidelines recommend annual assessment of urine albumin-to-creatinine ratio (UACR) and estimated glomerular filtration rate (eGFR) in all individuals with diabetes for screening and risk stratification of DN [3], 4]. However, in the early stages of DN, eGFR may remain within the normal range for a prolonged period, and albuminuria may demonstrate considerable biological variability and susceptibility to transient hemodynamic influences and clinical conditions [3], 5]. Therefore, there is an ongoing need for novel, feasible, and standardized biomarkers that may better reflect early glomerular barrier injury [5], [6], [7].

The pathogenesis of DN involves not only metabolic dysregulation but also endothelial dysfunction, loss of glomerular filtration barrier integrity, and podocyte injury [1], 7]. In the hyperglycemic milieu, the accumulation of advanced glycation end products (AGEs) and activation of the AGE–RAGE axis have been associated with cytoskeletal remodeling in endothelial cells and increased vascular permeability [8], 9]. In this context, moesin, a member of the ezrin/radixin/moesin (ERM) protein family that links the actin cytoskeleton to the plasma membrane, has emerged as a key mediator in the regulation of endothelial barrier function [10], 11]. Experimental data indicate that AGE exposure enhances moesin phosphorylation, thereby promoting endothelial permeability and cellular contraction [11], 12]. Moreover, cellular-level evidence suggests that moesin phosphorylation may represent a targetable signaling node in AGE-related endothelial barrier dysfunction [13]. Collectively, these findings raise the possibility that moesin may reflect biological processes associated with glomerular barrier disruption in DN.

Podocytes, another essential component of the glomerular filtration barrier, are structurally and functionally affected in the early stages of DN [7], 14]. Podocalyxin (PCX), a major sialomucin expressed on the apical surface of podocytes, plays a critical role in maintaining filtration barrier charge selectivity and podocyte architecture [14]. Increased urinary PCX excretion and the detection of podocyte-derived markers in urine have attracted attention as potential early indicators of DN [14], 15]. Nevertheless, the clinical standardization of urinary biomarkers is not always straightforward due to dilution effects, hydration status, collection variability, and, particularly, the challenges associated with 24-h urine sampling and patient compliance [14], 16]. For this reason, biomarker investigations based on serum samples – more easily accessible, rapid, and amenable to routine clinical evaluation – have gained increasing interest.

Although urinary PCX measurement may theoretically offer advantages in reflecting direct glomerular injury, a limited number of clinical studies have explored circulating PCX levels in relation to diabetic kidney disease parameters [17], 18]. It has been suggested that circulating PCX may not directly mirror renal processes alone but may instead reflect a broader axis of endothelial–podocyte injury. Therefore, rather than challenging the theoretical superiority of urinary PCX measurement, the present study adopts an exploratory approach, aiming to investigate whether serum levels may provide a clinically practical and biologically meaningful signal.

The rationale for the combined evaluation of moesin and PCX is based on targeting two complementary components of the glomerular barrier rather than focusing on a single cellular mechanism. While moesin may represent glomerular endothelial dysfunction through AGE-related cytoskeletal and permeability alterations [11], [12], [13], PCX is closely linked to podocyte integrity and filtration barrier charge selectivity [14]. Evaluating these two biomarkers within the same cohort may therefore allow a more integrated assessment of early microvascular barrier disruption in DN.

Accordingly, the aim of this study was to compare serum moesin and serum PCX levels (i) between individuals with type 2 diabetes mellitus (T2DM) and healthy controls, and (ii) between diabetic patients with and without microalbuminuria (early-stage DN), in order to explore their potential as candidate biomarkers.

## Participants and methods

### Study design

This study was designed as a single-center, cross-sectional, and exploratory investigation. The study population consisted of patients attending the Endocrinology and Metabolism Clinic of Konya City Hospital and healthy volunteers. All biochemical analyses were performed in the hospital’s central laboratory.

### Study groups

A total of 63 patients with T2DM with a disease duration of at least five years and 27 healthy controls (CG), aged between 18 and 65 years, were included in the study.

Diabetic participants were categorized according to their urine albumin-to-creatinine ratio (UACR) into normoalbuminuric and microalbuminuric groups. The study groups were defined as follows:

Control Group (CG): Healthy individuals without diabetes or known chronic disease (n=27).

DM Group: Normoalbuminuric diabetic patients (spot UACR <30 mg/g) (n=32).

DNP Group: Diabetic patients with early-stage diabetic nephropathy (microalbuminuria), defined as spot UACR 30–300 mg/g (n=31).

### Exclusion criteria

To minimize potential confounding factors that could influence circulating moesin and podocalyxin levels, individuals with chronic inflammatory, autoimmune, or systemic diseases (including thyroid disorders, rheumatologic diseases, and chronic liver disease), uncontrolled hypertension, advanced cardiovascular disease, malignancy, diabetic patients with macrovascular complications, pregnancy or lactation, renal replacement therapy, spot UACR >300 mg/g, acute infection, or age outside the predefined range (18–65 years) were excluded from the study. These conditions were excluded because systemic inflammation, oxidative stress, and endothelial dysfunction may independently affect circulating cytoskeletal proteins and glycoproteins. All participants were additionally screened for urinary tract infection, and none tested positive.

### Data collection

#### Anthropometric measurements

Height (cm), body weight (kg), body mass index (BMI, kg/m^2^), and waist circumference (cm) were measured in the morning after overnight fasting and with an empty bladder. All measurements were performed by the same trained healthcare personnel according to a standardized protocol.

#### Biochemical analyses

All venous blood samples were collected from the antecubital vein between 08:00 and 09:00 AM without the use of a tourniquet. Fasting duration was standardized to a minimum of 8 h. To prevent prolonged fasting-related hypoglycemia, oxidative stress alterations, and potential effects on biomarker levels in diabetic participants, fasting longer than 10 h was not permitted [19].

Biochemical parameters, including fasting glucose, blood urea nitrogen (BUN), creatinine, albumin, alanine aminotransferase (ALT), and lipid profile (triglycerides, total cholesterol, High-Density Lipoprotein Cholesterol (HDL-c), Low-Density Lipoprotein Cholesterol [LDL-c]), were measured using standard photometric methods on a Roche Cobas c501 analyzer (Roche Diagnostics, Germany). Glycated Hemoglobin (HbA1c) levels were determined using high-performance liquid chromatography (HPLC) (Adams HbA1c 8180v, Japan). Spot urine albumin-to-creatinine ratio was measured using an immunoturbidimetric method for albumin and an enzymatic colorimetric method for creatinine. Estimated glomerular filtration rate (eGFR) was calculated using the chronic kidney disease epidemiology collaboration equation (CKD-EPI equation).

#### Measurement of serum moesin and podocalyxin

Serum moesin levels were measured using a commercially available Human Moesin (MSN) ELISA kit (Cat. No: E4441Hu, BT Lab, China), and serum podocalyxin levels were measured using a Human Podocalyxin (PCX) ELISA kit (Cat. No: E1834Hu, BT Lab, China). All assays were performed according to the manufacturer’s instructions.

#### Outcomes

The primary outcome was the comparison of serum moesin and podocalyxin levels between healthy controls and diabetic groups (DM and DNP). The secondary outcome was the comparison of these biomarkers between diabetic patients with normoalbuminuria and those with microalbuminuria (DM vs. DNP).

#### Sample size

Participants who met the inclusion criteria and agreed to participate during the study period were consecutively enrolled. The study was designed as an exploratory investigation to evaluate the potential clinical relevance of the selected biomarkers. Given its exploratory nature, the sample size may be limited for extensive multivariable modeling, and this limitation was acknowledged in the interpretation of the findings.

### Statistical analysis

Statistical analyses were performed using IBM SPSS Statistics version 29 (IBM Corp., Armonk, NY). The distribution of variables was assessed using the Shapiro–Wilk test. As most variables were not normally distributed, non-parametric tests were used. Between-group comparisons were conducted using the Mann–Whitney U test for two-group comparisons and the Kruskal–Wallis test for three-group comparisons. Categorical variables were compared using the chi-square test or Fisher’s exact test, where appropriate. Because the control group differed significantly in age, serum moesin and podocalyxin levels were additionally analyzed as age-adjusted predicted values derived from regression models. Multiple linear regression analysis was used to determine independent predictors of serum moesin and podocalyxin levels. Binary logistic regression analysis was performed to evaluate associations with the presence of diabetic nephropathy. To reduce dimensionality and explore clustering patterns among measured variables, exploratory factor analysis (principal component analysis, PCA) was performed. Derived factor scores were subsequently used in logistic regression models. A p-value <0.05 was considered statistically significant.

### Ethics approval and consent to participate

The study was conducted in accordance with the principles of the Declaration of Helsinki. Ethical approval was obtained from the Non-Interventional Research Ethics Committee of KTO Karatay University Faculty of Medicine (Approval No: 2024/19; Date: 28.06.2024). Participant recruitment and sample collection were conducted between August 2024 and December 2024. Written and verbal informed consent was obtained from all participants prior to enrollment. All data were anonymized to ensure confidentiality.

## Results

### Baseline characteristics

The demographic and treatment-related characteristics of the study population are presented in Table 1. The healthy control group was significantly younger than both diabetic groups (p<0.001). No significant age difference was observed between normoalbuminuric diabetes (DM) and diabetic nephropathy (DNP) groups. BMI differed significantly across the three groups (p<0.001). Both diabetic groups had higher BMI values compared to controls. In addition, patients with diabetic nephropathy had significantly higher BMI compared to normoalbuminuric DM patients.

**Table 1:** Baseline demographic and clinical characteristics of the study groups.

Parameter	CG (n=27)	DM (n=32)	DNP (n=31)	p-Value
Age, years	41 (37–44)	56 (49–61)^b^	58 (50–61)^b^	<0.001
Gender, male/female, n (%)	11/16 (41/59)	14/18 (44/56)	12/19 (39/61)	0.920
BMI, kg/m^2^	27 (25–31)	31 (27–33)^a^	33 (31–38)^b,c^	<0.001

**Antidiabetic therapy**				

Metformin (no/yes), n (%)	–	4/28 (12/82)	5/26 (16/84)	0.479
DPP-4 inhibitors (no/yes), n (%)	–	17/15 (53/47)	13/18 (42/58)	0.262
SGLT2 inhibitors (no/yes), n (%)	–	18/14 (56/44)	16/15 (52/48)	0.454
Insulin (no/yes), n (%)	–	18/14 (56/44)	14/17 (45/55)	0.265

Data are presented as median (interquartile range) for continuous variables and number (percentage) for categorical variables. Continuous variables were compared using the Kruskal–Wallis test (with post-hoc pairwise Mann–Whitney U test where appropriate). Categorical variables were compared using the Chi-square test. ^a^p<0.05 vs. CG, ^b^p<0.001 vs. CG, ^c^p<0.05 vs. DM. BMI, body mass index; CG, control group; DM, normoalbuminuric diabetes mellitus; DNP, diabetic nephropathy with microalbuminuria; DPP-4, dipeptidyl peptidase-4; SGLT2, sodium–glucose cotransporter-2.

Gender distribution did not differ among the three groups (p=0.920). Among diabetic patients, the distribution of antidiabetic therapies, including metformin, DPP-4 inhibitors, SGLT2 inhibitors, and insulin use, did not significantly differ between DM and DNP groups. The age and BMI differences between the control and diabetic groups are considered when interpreting subsequent between-group comparisons.

### Biochemical and hematological comparison between DM and DNP groups

A comprehensive comparison of biochemical and hematological parameters between DM and DNP groups is presented in Table 2. Patients with diabetic nephropathy had significantly higher microalbuminuria levels (p<0.001) and higher HbA1c values (p=0.012) compared to DM patients without nephropathy. Serum albumin concentrations were significantly lower in the DNP group (p=0.044). No statistically significant differences were observed between the two diabetic groups in creatinine, urea, uric acid, eGFR, CRP, fasting glucose, LDL-C, HDL-C, triglycerides, ALT, hemoglobin, white blood cell count, or platelet count. Although median glucose levels were numerically higher in the DNP group, this difference did not reach statistical significance (p=0.063).

**Table 2:** Comparison of biochemical hematological parameters between normoalbuminuric diabetes (DM) and diabetic nephropathy (DNP) groups.

Parameter	DM (n=32)	DNP (n=31)	p-Value
Microalbuminuria (spot urine, mg/dL)	8.3 (4.0–13.0)	80 (44–176)	<0.001
HbA1c, %	7.55 (6.55–9.65)	8.50 (7.75–10.70)	0.012
Albumin, g/L	46.0 (44.0–47.5)	44.0 (42.0–47.0)	0.044
Creatinine, mg/dL	0.70 (0.60–0.80)	0.70 (0.60–0.90)	0.539
Urea, mg/dL	12 (10–16)	15 (11–18)	0.122
Uric acid, mg/dL	4.9 (3.9–5.8)	4.7 (3.9–5.7)	0.695
eGFR (CKD-EPI), mL/min/1.73 m^2^	99 (87–104)	95 (82–104)	0.251
CRP, mg/L	3.1 (1.7–5.4)	3.6 (1.7–10.5)	0.329
Glucose, mg/dL	131 (113–148)	168 (120–223)	0.063
LDL-C, mg/dL	115 (92–132)	113 (93–150)	0.695
HDL-C, mg/dL	43.5 (35.5–51.0)	42.0 (39.0–49.0)	0.962
TG, mg/dL	184 (133–243)	197 (114–275)	0.978
ALT, U/L	20 (16–28)	20 (14–25)	0.331
HGB, g/dL	14.5 (13.3–16.3)	14.0 (12.8–15.5)	0.449
WBC, ×10^3^/µL	8.05 (7.25–9.20)	9.10 (7.45–10.45)	0.146
PLT, ×10^3^/µL	251 (216–303)	284 (243–346)	0.124

Data are presented as median (interquartile range). Comparisons between groups were performed using the Mann–Whitney U test. DM, normoalbuminuric diabetes mellitus; DNP, diabetic nephropathy with microalbuminuria; HbA1c, glycated hemoglobin; eGFR, estimated glomerular filtration rate; CKD-EPI, chronic kidney disease epidemiology collaboration; CRP, c-reactive protein; LDL-C, low-density lipoprotein cholesterol; HDL-C, high-density lipoprotein cholesterol; TG, triglyceride; ALT, alanine aminotransferase; HGB, hemoglobin; WBC, white blood cell count; PLT, platelet count.

### Serum moesin and podocalyxin levels

Serum moesin and podocalyxin levels across the three study groups are presented in Table 3. Median moesin levels were significantly lower in both diabetic groups compared to the control group (overall p<0.001). Pairwise comparisons showed that moesin concentrations were reduced in DM and DNP groups relative to controls. A modest but statistically significant difference was observed between DM and DNP groups in the unadjusted analysis, with slightly higher moesin levels in the DNP group. Similarly, podocalyxin levels were significantly lower in both diabetic groups compared to controls (overall p<0.001). However, no significant difference was observed between normoalbuminuric DM and DNP groups in the primary comparisons.

**Table 3:** Age-adjusted median serum moesin and podocalyxin levels across study groups.

Parameter	CG (n=27)	DM (n=32)	DNP (n=31)	p-Value
Moesin, ng/L	2.332 (2.018–2.558)	1.227^b^ (1.100–1.468)	1.332^b,c^ (1.263–1.575)	<0.001
Adjusted predicted moesin, ng/L	1.958 (1.858–2.104)	1.517^b^ (1.362–1.720)	1.451^b^ (1.316–1.685)	<0.001
Podocalyxin, ng/mL	6.38 (4.50–7.84)	3.77^b^ (3.24–4.32)	3.65^a^ (3.24–4.49)	<0.001
Adjusted predicted podocalyxin, ng/mL	5.51 (5.20–5.84)	4.26^b^ (3.86–4.81)	4.08^b^ (3.71–4.73)	<0.001

Data are presented as median (interquartile range). Overall group comparisons were performed using the Kruskal–Wallis test. Pairwise post-hoc comparisons were conducted using the Mann–Whitney U test with appropriate correction. Adjusted predicted values were obtained from age-adjusted regression models. ^a^p<0.01 vs. CG, ^b^p<0.001 vs. CG, ^c^p<0.05 vs. DM. CG, control group; DM, normoalbuminuric diabetes mellitus; DNP, diabetic nephropathy with microalbuminuria.

Because the control group was significantly younger than both diabetic groups, age-adjusted analyses were performed using regression-derived predicted values. After adjustment for age, both moesin and podocalyxin levels remained significantly lower in diabetic patients compared to controls. However, the difference between DM and DNP groups was no longer statistically significant following adjustment.

Given the cross-sectional design and the relatively limited sample size, age-adjusted analyses are interpreted cautiously as exploratory approaches rather than definitive evidence of independent group differences.

In order to check the two new parameters as nephropathy status predictors, we have implemented binary logistic regression analysis, but neither moesin nor podocalyxin was a significant predictor of nephropathy status among DM patients.

To identify variables independently associated with serum moesin levels among diabetic patients, multiple linear regression analysis was performed (Table 4). Age was inversely associated with moesin concentrations (B=−28.0, p<0.001), indicating lower moesin levels with increasing age. Serum creatinine showed a positive association with moesin (B=1.069, p=0.001). Triglyceride levels, ALT, and white blood cell count were independently and inversely associated with moesin concentrations (p=0.014, p=0.048, and p=0.021, respectively). HDL-C did not reach statistical significance in the adjusted model (p=0.091). The final model explained approximately 38 % of the variance in serum moesin levels (adjusted R^2^=0.382). These findings suggest that age, renal function, and selected metabolic and inflammatory parameters are associated with circulating moesin levels in patients with diabetes mellitus.

**Table 4:** Independent predictors of serum moesin levels in patients with diabetes mellitus.

Predictor	B (95 % CI)	SE	p-Value
Age, years	−28.0 (−39.5 to −16.4)	5.79	<0.001
Creatinine, mg/dL	1.069 (435–1.703)	318.7	0.001
HDL-C, mg/dL	2.66 (−0.43 to 0.75)	1.55	0.091
TG, mg/dL	−0.88 (−1.58 to −0.18)	0.35	0.014
ALT, U/L	−8.81 (−17.51 to −0.09)	4.38	0.048
WBC, ×10^3^/µL	−64.8 (−119.6 to −9.9)	27.6	0.021

Adjusted R^2^=0.382. Multiple linear regression analysis was performed to identify independent predictors of serum moesin levels in patients with diabetes mellitus. Data are presented as regression coefficient (B) with 95 % confidence interval (CI) and standard error (SE). HDL-C, high-density lipoprotein cholesterol; TG, triglyceride; ALT, alanine aminotransferase; WBC, white blood cell count.

Multiple linear regression analysis was performed to explore variables independently associated with serum podocalyxin levels among diabetic patients (Table 5). Age was inversely associated with podocalyxin concentrations (B=−0.102, p<0.001). ALT and hemoglobin levels were also independently and negatively associated with podocalyxin (p=0.013 and p=0.034, respectively). Although creatinine showed a positive coefficient, this association did not reach statistical significance in the adjusted model (p=0.070). The final model explained approximately 27 % of the variance in serum podocalyxin levels (adjusted R^2^=0.271). These findings indicate that age and selected biochemical parameters are associated with circulating podocalyxin concentrations in patients with diabetes mellitus.

**Table 5:** Independent predictors of serum podocalyxin levels in patients with diabetes mellitus.

Predictor	B (95 % CI)	SE	p-Value
Age, years	−0.102 (−0.141 to −0.062)	0.020	<0.001
Creatinine, mg/dL	2.005 (−0.166 to 4.175)	1.092	0.070
ALT, U/L	−0.039 (−0.070 to −0.008)	0.016	0.013
HGB, g/dL	−0.229 (−0.439 to −0.018)	0.106	0.034

Adjusted R^2^=0.271. Multiple linear regression analysis was performed to determine independent predictors of serum podocalyxin levels in patients with diabetes mellitus. Data are presented as regression coefficient (B) with 95 % confidence interval (CI) and standard error (SE). ALT, alanine aminotransferase; HGB, hemoglobin.

### Factor analysis

Given the number of clinical and biochemical parameters measured, exploratory factor analysis was performed to identify potential clustering patterns among variables (Table 6). Principal component extraction with varimax rotation was applied. The Kaiser–Meyer–Olkin measure of sampling adequacy (KMO=0.617) and Bartlett’s test of sphericity (p<0.001) supported the appropriateness of the analysis.

**Table 6:** Factor analysis of clinical and biochemical parameters in patients with diabetes mellitus.

Factor	Variables included after rotation	Factor loading	Explained variance, %
Factor 1	Moesin, ng/L	−0.748	18
	Podocalyxin, ng/mL	−0.621	
	Age, years	0.615	
	WBC, ×10^3^/µL	0.602	
	Microalbuminuria (spot urine, mg/dL)	0.512	
Factor 2	Gender	−0.843	16
	HGB, g/dL	0.696	
	Creatinine, mg/dL	0.603	
	ALT, U/L	0.588	
Factor 3	TG, mg/dL	0.732	15
	HDL-C, mg/dL	0.718	
	Glucose, mg/dL	0.640	
	LDL-C, mg/dL	0.598	

Total explained variance of the model: 49 %. Exploratory factor analysis was performed using principal component extraction with varimax rotation. Factor loadings ≥0.50 were considered significant for factor interpretation. WBC, white blood cell count; HGB, hemoglobin; ALT, alanine aminotransferase; TG, triglyceride; HDL-C, high-density lipoprotein cholesterol; LDL-C, low-density lipoprotein cholesterol.

Three components were extracted, explaining a total of 49 % of the variance.

Factor 1 accounted for 18 % of the variance and included moesin, podocalyxin, age, white blood cell count, and microalbuminuria.

Factor 2 explained 16 % of the variance and consisted of gender, hemoglobin, creatinine, and ALT.

Factor 3 accounted for 15 % of the variance and included triglycerides, HDL-C, glucose, and LDL-C.

Only factor loadings ≥0.50 were considered for interpretation.

These findings suggest that circulating moesin and podocalyxin levels cluster with age, inflammatory markers, and microalbuminuria rather than forming an isolated biomarker dimension. However, given the exploratory nature of the analysis and the limited sample size, these clustering patterns should be interpreted cautiously.

Factor scores derived from exploratory factor analysis were subsequently entered into a binary logistic regression model to examine their association with the presence of diabetic nephropathy among diabetic patients (Table 7). Among the extracted components, only Factor 1 showed a statistically significant association with nephropathy status (OR=4.03, 95 % CI: 1.42–11.47, p=0.009). Factors 2 and 3 were not significantly associated with nephropathy. Factor 1 included moesin, podocalyxin, age, white blood cell count, and microalbuminuria. The observed association suggests that this cluster of variables may reflect a broader clinical–biological profile linked to nephropathy status. However, given the cross-sectional design and limited sample size, these findings should be interpreted cautiously and cannot be considered evidence of predictive or causal relationships.

**Table 7:** Binary logistic regression analysis of factor scores for the presence of diabetic nephropathy.

Variable	B, SE	Wald	OR (95 % CI)	p-Value
Factor 1	0.40 (0.53)	6.85	4.03 (1.42–11.47)	0.009
Factor 2	−0.04 (0.25)	0.03	0.96 (0.58–1.57)	0.865
Factor 3	0.47 (0.33)	1.97	1.60 (0.83–3.06)	0.160

Binary logistic regression analysis was performed to evaluate the association between factor scores derived from exploratory factor analysis and the presence of diabetic nephropathy. Data are presented as regression coefficient (B) with standard error (SE), odds ratio (OR) with 95 % confidence interval (CI), and Wald statistics.

## Discussion

In this study, serum moesin and serum podocalyxin levels were evaluated in individuals with type 2 diabetes mellitus. Although significant differences were observed between diabetic participants and healthy controls, neither biomarker demonstrated discriminatory performance between DM and DNP groups. While this finding may appear clinically “negative,” it carries an important implication in the context of biomarker development: serum moesin and serum PCX may not serve as reliable tools for distinguishing early diabetic nephropathy, or at least may require more refined study designs and combined measurement matrices (e.g., simultaneous serum and urine assessment) to demonstrate such potential.

Current clinical guidelines continue to rely on UACR and eGFR for screening and risk stratification of DKD [3], 4]. However, eGFR may remain within the normal range for a prolonged period during early disease stages, and albuminuria exhibits biological variability and sensitivity to transient clinical conditions. Consequently, the search for novel biomarkers remains ongoing. Importantly, the ability of a biomarker to distinguish a clinical phenotype such as early DN is determined not only by its pathophysiological relevance but also by measurement matrix, disease stage, confounding factors, and sample size considerations. In our study, the absence of differentiation between DM and DNP groups may partly reflect the positioning of the participants within the early DN window, defined by microalbuminuria. Microalbuminuria does not always represent a linear progression toward structural glomerular damage and may regress or fluctuate in certain individuals [3], 4]. Therefore, the lack of clear separation at the serum level may be biologically plausible.

Hyperglycemia-induced oxidative stress, accumulation of AGEs, and endothelial dysfunction are central mechanisms in the pathogenesis of diabetic complications [20], 21]. Activation of the AGE–RAGE axis promotes reactive oxygen species generation and inflammatory signaling, affecting cytoskeletal organization and vascular structural proteins [20]. Moesin, a member of the ERM family, regulates the linkage between the cytoskeleton and plasma membrane and plays a key role in endothelial barrier integrity [10], 12], 22]. Podocalyxin is a negatively charged transmembrane glycoprotein expressed on podocytes and contributes to the maintenance of glomerular filtration barrier charge selectivity and structural stability [23]. From a biological perspective, alterations in both proteins under diabetic conditions are plausible.

In our study, both serum moesin and podocalyxin levels were lower in diabetic individuals compared to healthy controls, suggesting that diabetes may be associated with systemic structural and vascular remodeling processes. However, the absence of significant differences between normoalbuminuric and microalbuminuric diabetic patients indicates that these markers may not adequately reflect kidney-specific injury at the serum level during early disease stages.

The interpretation of serum PCX findings must consider the serum–urine matrix distinction. PCX has predominantly been investigated in urine, where it has been proposed as an early indicator of podocyte injury [15], [23], [24], [25], [26]. Data regarding circulating PCX levels remain limited [17], 18]. While serum measurement offers practical advantages in routine clinical settings, it may not directly capture localized glomerular shedding or podocyte detachment to the same extent as urinary measurement. The absence of urinary PCX assessment in our study represents an important methodological limitation. It is possible that serum PCX reflects a broader endothelial–podocyte injury axis rather than direct glomerular barrier disruption. Furthermore, data from biopsy-confirmed DKD cohorts suggest that tissue and urinary PCX may behave differently, and renal tissue expression does not always correspond to urinary excretion patterns [27]. This variability underscores that PCX biology cannot be generalized across a single biological matrix. Therefore, our findings suggest that serum PCX alone may have limited discriminatory value in early DN and that combined serum and urinary assessment – preferably in longitudinal designs – may provide more meaningful insights.

Regarding moesin, literature evidence has demonstrated that moesin phosphorylation is involved in AGE-mediated endothelial barrier dysfunction and increased vascular permeability [9], 11], 12]. Regarding moesin, experimental evidence has demonstrated that moesin phosphorylation is involved in AGE-mediated endothelial barrier dysfunction and increased vascular permeability [9], 11], 12], 28]. AGEs can induce cytoskeletal rearrangement and endothelial hyperpermeability, processes in which moesin phosphorylation plays a regulatory role [11], 12]. However, endothelial dysfunction is a systemic phenomenon that may develop early in diabetes and is influenced by obesity, insulin resistance, and dyslipidemia. Consequently, alterations in circulating moesin levels may reflect systemic endothelial remodeling rather than kidney-specific injury. This may explain why moesin levels differed between diabetic and control groups but failed to distinguish DNP from DM. In other words, moesin may represent a marker of diabetes-associated vascular and cytoskeletal dysregulation without sufficient specificity for early nephropathy.

In multivariable regression analyses, age emerged as the most consistent determinant of both moesin and podocalyxin levels. This finding suggests that circulating cytoskeletal and glycoprotein structures are influenced not only by renal function but also by systemic aging processes and low-grade inflammation. In exploratory factor analysis, moesin and podocalyxin clustered together with age, white blood cell count, and microalbuminuria within the same component. This pattern supports the concept that these biomarkers may belong to a broader inflammation–vascular injury axis rather than representing isolated renal markers. Nevertheless, the exploratory nature of this analysis requires validation in independent cohorts.

Several strengths and limitations must be considered. A strength of this study is the simultaneous evaluation of two biologically complementary candidate biomarkers in a clinically relevant phenotypic framework based on UACR and eGFR. However, important limitations exist. The control group was not fully matched in terms of age and BMI, and although age-adjusted analyses were performed, residual confounding cannot be excluded, particularly given the modest sample size. The cross-sectional design precludes causal inference and does not allow assessment of longitudinal biomarker dynamics or prediction of disease progression. Additionally, the phosphorylation status of moesin was not evaluated. Given that moesin biological activity is closely linked to its phosphorylation state, total serum levels may not fully reflect functional activation [22].

Considering these limitations, our findings should be interpreted as hypothesis-generating rather than definitive. Future studies should (i) evaluate serum and urinary PCX concurrently, (ii) include larger and better-matched cohorts, and (iii) adopt longitudinal designs to assess predictive value for disease progression. The DKD biomarker literature consistently emphasizes the necessity of independent validation and robust methodological frameworks before clinical implementation [6], 14]. In this context, our study contributes to the field by clarifying that serum moesin and serum PCX demonstrate limited discriminatory performance for early DN, thereby helping refine future research directions.

## Conclusions

In conclusion, serum moesin and podocalyxin levels were lower in patients with type 2 diabetes mellitus compared with healthy controls; however, neither biomarker independently discriminated early-stage diabetic nephropathy. These findings suggest that both molecules may reflect diabetes-related systemic vascular and inflammatory alterations rather than serving as specific indicators of early renal involvement. At the current stage, their utility as standalone biomarkers for early nephropathy appears limited. Larger-scale, prospective studies incorporating combined serum and urinary measurements are warranted to further clarify their clinical relevance.
